# Heterologous prime-boost vaccination with DNA and MVA vaccines, expressing HIV-1 subtype C mosaic Gag virus-like particles, is highly immunogenic in mice

**DOI:** 10.1371/journal.pone.0173352

**Published:** 2017-03-09

**Authors:** Ros Chapman, Tsungai Ivai Jongwe, Nicola Douglass, Gerald Chege, Anna-Lise Williamson

**Affiliations:** 1 Institute of Infectious Disease and Molecular Medicine and Division of Medical Virology, Department of Pathology, Faculty of Health Sciences, University of Cape Town, Cape Town, South Africa; 2 National Health Laboratory Services, Groote Schuur Hospital, Cape Town, South Africa; University of Massachusetts Medical School, UNITED STATES

## Abstract

In an effort to make affordable vaccines suitable for the regions most affected by HIV-1, we have constructed stable vaccines that express an HIV-1 subtype C mosaic Gag immunogen (BCG-Gag^M^, MVA-Gag^M^ and DNA-Gag^M^). Mosaic immunogens have been designed to address the tremendous diversity of this virus. Here we have shown that Gag^M^ buds from cells infected and transfected with MVA-Gag^M^ and DNA-Gag^M^ respectively and forms virus-like particles. Previously we showed that a BCG-Gag^M^ prime MVA-Gag^M^ boost generated strong cellular immune responses in mice. In this study immune responses to the DNA-Gag^M^ and MVA-Gag^M^ vaccines were evaluated in homologous and heterologous prime-boost vaccinations. The DNA homologous prime boost vaccination elicited predominantly CD8+ T cells while the homologous MVA vaccination induced predominantly CD4+ T cells. A heterologous DNA-Gag^M^ prime MVA-Gag^M^ boost induced strong, more balanced Gag CD8+ and CD4+ T cell responses and that were predominantly of an effector memory phenotype. The immunogenicity of the mosaic Gag (Gag^M^) was compared to a naturally occurring subtype C Gag (Gag^N^) using a DNA homologous vaccination regimen. DNA-Gag^N^ expresses a natural Gag with a sequence that was closest to the consensus sequence of subtype C viruses sampled in South Africa. DNA-Gag^M^ homologous vaccination induced cumulative HIV-1 Gag-specific IFN-γ ELISPOT responses that were 6.5-fold higher than those induced by the DNA-Gag^N^ vaccination. Similarly, DNA-Gag^M^ vaccination generated 7-fold higher levels of cytokine-positive CD8+ T cells than DNA-Gag^N^, indicating that this subtype C mosaic Gag elicits far more potent immune responses than a consensus-type Gag. Cells transfected and infected with DNA-Gag^M^ and MVA-Gag^M^ respectively, expressed high levels of Gag^M^ and produced budding virus-like particles. Our data indicates that a heterologous prime boost regimen using DNA and MVA vaccines expressing HIV-1 subtype C mosaic Gag is highly immunogenic in mice and warrants further investigation in non-human primates.

## Introduction

Despite the reduction of deaths associated with HIV-1 infection and HIV-1-related illnesses over recent years [1], attributable to expansion of educational programs, use of condoms, male circumcision, as well as the use of anti-retroviral (ARV) drugs, the best long-term means of preventing the spread of this epidemic is a prophylactic HIV-1 vaccine.

One of the major difficulties faced in developing a successful HIV-1 vaccine is the enormous diversity of the virus. One approach used to overcome this problem is the use of mosaic immunogens which have been computationally designed to specifically overcome this hurdle by maximizing the inclusion of common T cell epitopes. When compared to consensus immunogens, both full length and conserved region polyvalent mosaic immunogens of HIV-1 group M have shown increased breadth and depth of antigen-specific T-cell responses [2–5]. Studies have also shown that mosaic HIV-1 Gag antigens are processed and presented by peripheral blood mononuclear cells (PBMCs) derived from HIV-1-infected individuals [6]. An HIV-1 subtype C mosaic *gag* gene was chosen in this study as subtype C is the predominant subtype in sub-Saharan Africa.

Researchers in the HIV vaccine field have been trying to improve upon the 31% protection generated in the RV144 Trial which used a recombinant canary poxvirus prime and a gp120 protein boost [7]. Various groups have shown that a heterologous DNA prime, poxvirus boost considerably improves the immune response as compared to homologous poxvirus vaccination regimens [8–11]. In addition DNA vaccines are relatively easy and affordable to produce, thermostable, safe with no risk of virulence or anti-vector immunity and multiple plasmids can be mixed and used as a broad spectrum vaccine. The use of a porcine circovirus enhancer element upstream of the *gag* gene in the DNA vaccines used in this study allows for dose sparing due to increased expression of Gag [12].

High magnitude, polyfunctional CD8+ T cell responses to Gag have been shown to be an important component of the control of viremia in HIV positive long term non-progressors and elite controllers. Therefore Gag is considered to be an important component of HIV vaccines as a strong T cell response could result in early clearance of HIV infected cells via CD8+ T cells at the site of infection, control of spread from the entry portal and control of viremia [13]. Many candidate HIV vaccines express a number of HIV proteins, however this can result in a poor immune response to Gag as other proteins may be immunodominant. This was seen in the South African Aids Vaccine Initiative DNA-C2 and MVA-C vaccines which expressed two immunogens, a polyprotein consisting of Gag, reverse transcriptase, Tat and Nef (Grttn) and a truncated envelope (Env). When tested in clinical trial the dominant response to these vaccines was a CD4+ T cell response to Env with fairly low Gag CD4+ and very weak Gag CD8+ T cell responses [14]. Similarly in the EV02 phase I trial a prime boost regimen consisting of DNA and NYVAC vectors expressing Env and a polyprotein containing Gag, Pol and Nef primarily induced CD4+ T cell responses against Env [11]. Thus in this study Gag was expressed alone and not as a polyprotein.

Previously we showed that a low dose of MVA expressing an HIV-1 subtype C mosaic Gag (10^4^ pfu) could effectively boost a BCG prime expressing the same immunogen, generating strong, cellular immune responses against Gag in mice [15]. In this paper we show that this computationally generated HIV-1 subtype C mosaic Gag has the properties of a normal HIV-1 Gag protein, is able to form virus-like particles (VLPs) and is more immunogenic than a natural Gag protein. A heterologous DNA prime-MVA boost regimen generated significantly improved T cell responses than homologous vaccination with either the DNA or MVA vaccines.

## Methods

### Construction of DNA vaccines

The plasmid pTHpCapR contains the commonly used pTH DNA vaccine vector backbone [16] and has a porcine circovirus enhancer element upstream of the cytomegalovirus (CMV) AD169 immediate-early promoter to facilitate increased expression of the immunogen, thus enabling dose sparing of the vaccine [12]. In this study, three DNA vaccines were constructed using the pTHpCapR backbone. The vaccine vector, DNA^E^, was made for use as an empty vector negative control. DNA-Gag^M^ contains the HIV-1 subtype C *gag* mosaic insert [15], and DNA-Gag^N^ the natural HIV-1 strain Du422 subtype C *gag* gene. The Gag protein from strain Du422, was chosen as it has the closest sequence similarity to the South African consensus sequence [17].

The pTHpCapR plasmid backbone [12] was used to construct the three DNA vaccines used in this paper. The full length HIV-1 subtype C mosaic [18] and HIV-1 Du422 *gag* genes were codon optimised for expression in humans and cloned into the EcoRI and HindIII sites of pTHpCapR to generate DNA-Gag^M^ and DNA-Gag^N^ respectively. Plasmid DNA^E^ contains no insert and was constructed by blunting the ends of plasmid pTHpCapR that had been digested with EcoRI and HindIII and re-ligating. Large-scale amplification of DNA constructs for immunization was carried out by Aldevron (Fargo, ND); purified plasmid DNA was formulated in water.

### Recombinant MVA expressing subtype C mosaic Gag (MVA-Gag^M^)

The HIV-1 subtype C mosaic *gag* was inserted between open reading frames A11R and A12L of the MVA virus genome under the transcriptional control of the modified-H5 promoter. MVA-Gag^M^ virus stock was prepared and titrated as previously described [15].

### Cell culture

HEK 293T and BHK-21 cells (ATCC, Manassas, VA) were maintained in Dulbecco’s modified Eagle’s medium (DMEM; Gibco-Invitrogen) supplemented with 5 or 10% Foetal Bovine Serum (FBS) and antibiotics (penicillin and streptomycin) using standard cell culture procedures.

### Gag p24 ELISA

Transfections of HEK 293T cells (10^6^ cells/well in a 6-well tissue culture plate) were performed 24 hours post trypsinization when the cells were at 60 to 70% confluence, using 4μg DNA and 4μl X-treme Gene HP^®^ transfection reagent (Roche; Switzerland). Similarly, BHK-21 and HeLa cells (0.3x10^6^ cells/well) were infected with MVA at an MOI of 1 in 6-well tissue culture places, when 60–70% confluent and left to adsorb for 2 hours. The inoculum was then removed and fresh media added.

To investigate the kinetics of Gag p24 expression the supernatant of transfected or infected cells was removed and stored. Cells were then washed with PBS and lysed by addition of Glo lysis buffer (Promega; USA) containing a protease inhibitor (cOmplete EDTA-free^®^: Roche, Switzerland). Capture ELISA was carried out using the Elecsys HIV Ag^®^ (p24) kit (Roche; Switzerland).

### Electron microscopy

Cells transfected with DNA or infected with MVA vaccines were scraped into the cell culture medium, pelleted, washed with PBS and fixed with 2.5% glutaraldehyde in PBS pH 7.4 overnight at 4°C in the dark. Fixed cells were gently resuspended and pelleted, washed twice in PBS and resuspended in 2% low melting point agarose in PBS at 37°C. Samples were then incubated in 0.5% tannic acid for 1 hour and washed once in PBS prior to post-fixing with 1% osmium tetroxide in PBS, washing and dehydrating through an ethanol series. The samples were then incubated in a 1:1 acetone: resin mixture overnight, followed by a 1:3 acetone: resin mixture and then an overnight incubation in 100% resin. Ultrathin sections mounted on copper grids were stained with 2% uranyl acetate for 10 min, rinsed thoroughly in ultrapure water, post-stained with lead citrate for 10 min, rinsed and dried. Specimens were visualized with a FEI Tecnai 20 transmission electron microscope.

### Mice vaccinations

The animal used in this study were housed and maintained in accordance with the South African national guidelines for Use of Animals for Scientific Purposes (SANS Code 10386) which is also in line with EU Directive 2010/63/EU. Groups of four to five 6–8 weeks old female BALB/c mice were used for each experiment. The DNA and MVA vaccines were administered bilaterally into the tibialis muscle of each hind leg (50μl each) at a total dose of 10μg per DNA vaccine and 10^4^ pfu of MVA. The pre-determined experimental end point was 12 days after the final vaccination. The vaccination schedule and all the animal procedures were approved by the University of Cape Town Animal Research Ethics Committee (reference UCT AEC 12–059) and performed by a trained animal technologist.

### Immunogenicity assays to evaluate mosaic vaccines

At the experimental endpoint spleens from mice in each vaccination group were harvested and pooled. A single cell suspension from the spleens was prepared as previously described [19] for use in immunogenicity assays. The amino acid sequences of the peptides used were AMQMLKDTI (GagCD8^+^ peptide) and NPPIPVGRIYKRWIILGLNK (GagCD4(13) peptide) and FRDYVDRFFKTLRAEQATQE (GagCD4(17) peptide)(21–23).

IFN-γ ELISPOT assays were carried out as previously described [19]. The method used for intracellular cytokine staining and staining of cell surface molecules is described in the article by Burgers et al., 2006 [20]. The following antibodies were used; anti-CD3+ Alexa 700, anti-CD4+ PE-Cy7, anti-CD8+ APC-Cy7, anti-CD62L APC, and anti-CD44 FITC. The cytokine antibodies were all PE-conjugated (0.2μg anti-TNF-PE, 0.06 μg anti-IL-2-PE, 0.06 μg anti-IFN-γ-PE) and were obtained from BD Biosciences. The anti-CD4+ PE-Cy7, anti-CD8+ APC-Cy7 were also obtained from BD Biosciences. All the other antibodies were obtained from eBioscience.

### Statistical analysis

Data was statistically analysed using Prism version 5.0 (Graphpad Software, San Diego, CA). The *t* test for independent unpaired parametric comparisons was applied to assess the level of significance of comparisons between means. All tests were two-tailed. P values ≤ 0.05 were considered significant. The false discovery rate (FDR) step-down procedure described in the paper by Columb & Sgadai was used to correct for multiple comparisons [21].

## Results

### Confirmation of VLP production by DNA-Gag^M^, DNA-Gag^N^ and MVA-Gag^M^ vaccines

**Fig 1:**
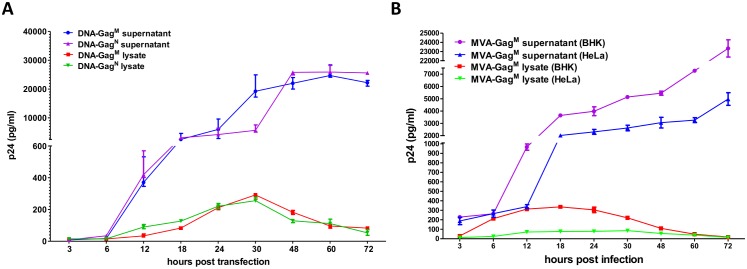
**p24 Gag production by:** (**A**) **DNA vaccines in HEK cells.** HEK cells were transfected with 4μg of DNA-Gag^M^ or DNA-Gag^N^ and samples taken at the indicated time points. (**B**) **Recombinant MVA in BHK-21 and HeLa cell lines.** Permissive (BHK-21) and non-permissive (HeLa) cell lines were infected at an MOI of 0.1 with MVA-Gag^M^ and samples taken at the indicated time points. Gag p24 was detected using an ELISA assay.

Gag p24 ELISAs of transfected HEK 293 cell-free lysates and supernatants were carried out at different time points post transfection to confirm Gag expression from the DNA vaccines (Fig 1A). p24 levels in the lysate of cells transfected with either DNA vaccine increased from 0–30 h.p.t, and started decreasing thereafter to levels below 100pg/ml at 72 h.p.t. Significantly more p24 was detected in the supernatant than the lysate from HEK 293 cells transfected with either of the DNA vaccines (Fig 1A). The expression of p24 in the cell supernatant was similar for both DNA vaccines over time, increasing from 6–48 hours post transfection (h.p.t.) to levels ≥20000pg/ml, and almost plateauing from 48–72 h.p.t. This high level of expression can be attributable to the porcine circovirus enhancer element.

For MVA-Gag^M^, the lysates and supernatants of infected permissive (BHK-21) and non-permissive (HeLa) cell lines were used to determine Gag expression by p24 ELISA (Fig 1B). Gag p24 production from cells infected with MVA-Gag^M^ was detected from as early as 3 hours post infection (h.p.i.) in the supernatants of both cell lines (Fig 1B) at values >100pg/ml. The amount of p24 increased over time in the cell supernatants, reaching >4000pg/ml at 72h.p.i in infected HeLa cells and >20000pg/ml in BHK-21 cells. BHK-21 cells support MVA replication, which explains the increase in Gag expression in this cell line. Detectable p24 decreased in the cell lysates between 24 and 72 h.p.i, dropping to levels below 100pg/ml. Overall, there was more p24 detected in the supernatants than lysates from both cell lines infected with MVA-Gag^M^ (Fig 1B).

**Fig 2:**
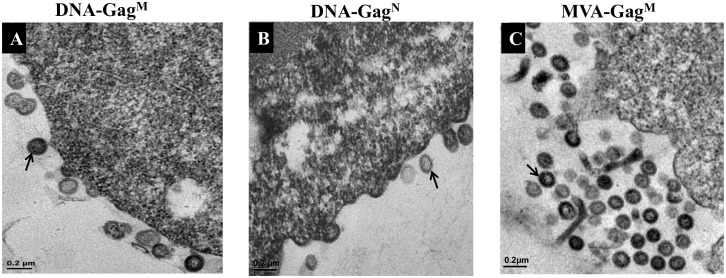
Electron micrographs of VLP formation in HEK293 cells transfected with (A) DNA-Gag^M^, (B) DNA-Gag^N^ and (C) BHK-21 cells infected with MVA-Gag^M^ 30 hours post transfection or infection. HEK293 cells were transfected with 4μg of the DNA vaccines and BHK-21 cells were infected with MVA-Gag^M^ at an MOI of 1. The VLPs are indicated by arrows. The scale bars represent 200nm.

The presence of p24 Gag in the supernatant of infected cells suggested the formation of virus-like particles (VLPs) so transmission electron microscopy (TEM) was carried out to confirm this. Extracellular VLPs as well as budding particles were detected in both DNA-Gag^M^ (Fig 2A) and DNA-Gag^N^ (Fig 2B) transfected HEK 293 cells. MVA-Gag^M^ produced VLPs in both BHK-21 cells which are permissive to MVA replication (Fig 2C) and HeLa cells which are not permissive to MVA replication (not shown).

### Immune responses in BALB/c mice elicited by DNA and MVA vaccines expressing a Gag^M^ immunogen

**Fig 3:**
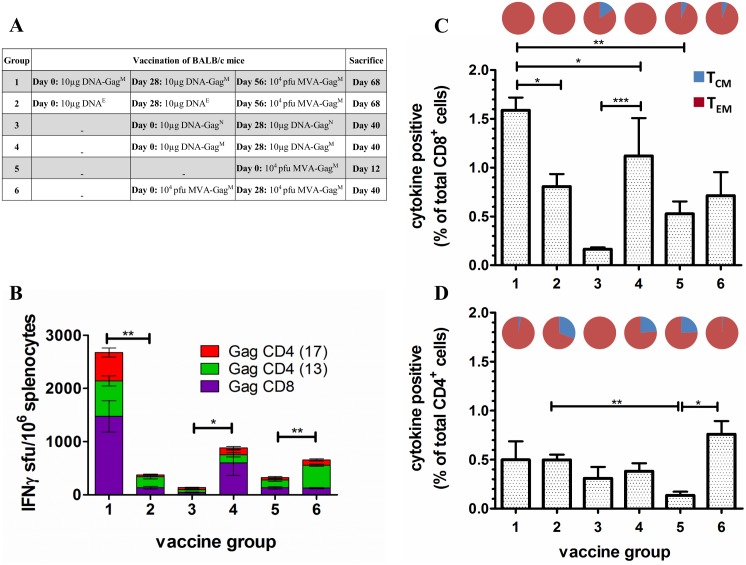
Immunological evaluation of a DNA-Gag^M^ prime/MVA-Gag^M^ boost in BALB/c mice. (**A**) Vaccination schedule used. (**B**) Cumulative IFN-γ ELISPOT CD8^+^ and CD4^+^ responses of vaccinated mice to HIV-1 Gag peptides. The ELISPOT assay was done using pooled spleens on the day of sacrifice using three Gag-specific peptides for stimulation. Bars are the mean and standard deviation of the mean responses for the indicated individual peptides from 3 independent experiments. Responses are expressed as sfu/10^6^ splenocytes after background subtraction. Horizontal bars with asterisks indicate statistical significance of the mean responses between the indicated groups. *p<0.05, **p<0.01, ***p<0.001; Student t-test of unpaired data. Total frequency of CD8+ (**C**) and CD4+ (**D**) T cells producing IFN-γ, IL-2, and/or TNF-α in response to HIV-1 Gag peptide stimulation. ICS and flow cytometry were carried out on pooled spleens per group using three Gag-specific peptides for stimulation. The memory distribution of the cytokine producing T-cells in the central and effector memory compartment (T_CM_ and T_EM_) are represented as pie charts above each corresponding bar per group. Cells were positive for cytokine production if the proportion was ≥ 0.05% after subtracting the background. The cellular phenotype was positive if there were ≥ 10 cells per test.

The immune responses elicited following the vaccine regimens shown in Fig 3A are graphically presented in Fig 3B–3D. The MVA-Gag^M^ vaccine significantly boosts two priming doses of DNA-Gag^M^ (p<0.01) as seen in the 3-fold increase in the mean cumulative Gag-specific IFN-γ ELISPOT responses between Group 1 mice (DNA-Gag^M^/MVA-Gag^M^) and Group 4 mice (DNA-Gag^M^/DNA-Gag^M^) that only received two priming does of DNA-Gag^M^ (Fig 3B; 882.3 ± 297.8 sfu/10^6^ splenocytes).

Mice that were vaccinated with two doses of DNA-Gag^M^ only (Fig 3B; Group 4) had mean cumulative Gag-specific IFN-γ ELISPOT responses that were 6.5–fold greater than mice vaccinated with two doses of DNA-Gag^N^ (Fig 3B; Group 3–135.7 ± 14 sfu/10^6^ splenocytes). These results indicate that the mosaic Gag is significantly more immunogenic than the natural Gag (p<0.05). Surprisingly, two doses of DNA-Gag^M^ (Group 4) elicited mean cumulative Gag-specific IFN-γ ELISPOT responses that were 1.3–fold greater than mice vaccinated with two doses of MVA-Gag^M^ (Fig 3B; Group 6–656.7 ± 8.5 sfu/10^6^ splenocytes). Mice vaccinated with two doses of DNA-Gag^M^ (Group 4) had higher responses to the CD8 than the CD4 Gag peptides (604 ± 239.2 sfu/10^6^ and 278.4 ± 32.6 sfu/10^6^ splenocytes respectively). Whereas mice vaccinated with two doses of MVA-Gag^M^ (Group 6) had more responses to the CD4 than the CD8 Gag peptide (530 ± 20.5 sfu/10^6^ and 126.7 ± 11.7 sfu/10^6^ splenocytes respectively).

There was a 7.1-fold significant difference (p<0.001) in the mean cumulative Gag-specific IFN-γ ELISPOT response of mock-primed mice boosted with 10^4^ pfu MVA-Gag^M^ (Fig 3B; Group 2–375 ± 70.7 sfu/10^6^ splenocytes) compared to mice primed with two doses of DNA-Gag^M^ and similarly boosted (Fig 3B; Group 1–2675.3 ± 292.8 sfu/10^6^ splenocytes). Group 2 mice had a predominantly CD4 response (241.7 ± 29.7 sfu/10^6^ splenocytes) to Gag, whereas a prime with DNA-Gag^M^ elicited a fairly balanced response to Gag CD4 and CD8 peptides (Group 1–1200.3 ± 183.2 sfu/10^6^ and 1475 ± 91.6 sfu/10^6^ splenocytes respectively). The efficiency of the DNA-Gag^M^ prime is also evident on comparison of the mean cumulative Gag-specific IFN-γ ELISPOT responses between Group 1 mice (2675.3±292.8 sfu/10^6^ splenocytes) and Group 5 mice that were only vaccinated with a single dose of MVA-Gag^M^ (323.7 ± 23.9 sfu/10^6^ splenocytes). There was an 8.3-fold significant difference between the groups (p<0.001). A comparison of the mean cumulative Gag-specific IFN-γ ELISPOT responses in Group 5 mice (MVA-Gag^M^) and Group 2 mice (DNA^E^/MVA-Gag^M^) that were mock-primed and boosted with MVA-Gag^M^ indicated that the mock-prime did not influence the immune response elicited by the MVA-Gag^M^ boost (Group 2–375 ± 70.4 sfu/10^6^ splenocytes). The mock DNA prime did, however, increase the CD4 response (Fig 3D).

The proportions of cytokine-producing T cells as well as the memory phenotypes induced were determined by flow cytometry (Fig 3C and 3D). All vaccine regimens elicited cytokine-producing CD8^+^ and CD4^+^ T cells. However, the proportion of CD8^+^ T cells producing cytokines was greater than that of CD4^+^ T cells for most of the regimens. Mice vaccinated with two doses of DNA-Gag^M^ (Group 4) elicited a notably higher CD8 response that those vaccinated with DNA-Gag^N^ (Group 3). Two doses of DNA-Gag^N^ (Group 3) and two doses of MVA-Gag^M^ (Group 6) elicited higher CD4 than CD8 responses.

A DNA-Gag^M^/MVA-Gag^M^ heterologous prime-boost vaccination elicited a cytokine-positive CD8^+^ T cell response that was 2-fold greater (p<0.05) than mock-primed mice that were similarly boosted (Fig 3C; Group 1—DNA-Gag^M^/MVA-Gag^M^—1.59%; Group 2—DNA^E^/MVA-Gag^M^—0.81% respectively). Both vaccine groups had cytokine-positive T cells with an effector memory phenotype. There was no significant difference in the proportion of cytokine-positive CD4^+^ T cells between the two groups (Fig 3D; Group 1–0.5%; Group 2–0.35%). However, Group 1 mice (DNA-Gag^M^/MVA-Gag^M^) had more cytokine-positive CD4^+^ T cells with an effector memory phenotype (97.5%) than Group 2 mice (DNA^E^/MVA-Gag^M^; 67%). Thus, two doses of the DNA-Gag^M^ vaccine significantly primed the MVA-Gag^M^ boost by increasing the proportion of cytokine-positive CD8^+^ T cells and the effector memory phenotype of cytokine-positive CD4^+^ T cells.

The efficiency of the MVA-Gag^M^ boost was determined by comparing immune responses from mice that received the DNA-Gag^M^/MVA-Gag^M^ heterologous prime-boost regimen (Group 1) to those vaccinated with the DNA-Gag^M^/DNA-Gag^M^ regimen (Group 4). Cytokine-positive CD8^+^ T cells from Group 1 (1.59%) mice were 1.4-fold significantly higher than those elicited by Group 4 mice (1.12%; Fig 3C). All the cytokine-positive CD8^+^ T cells had an effector memory phenotype in both groups. Surprisingly, there was only a 1.3-fold difference in cytokine-positive CD4^+^ T cells in mice that received the heterologous prime-boost vaccination (Group 1–0.49%) compared to those that received a homologous DNA prime-boost vaccination (Group 4–0.38%; Fig 3D). However the effector memory phenotype increased from 76.7% (Group 4) to 97.5% (Group 1) following the boost vaccination. Thus the MVA-Gag^M^ boost vaccination increases cytokine-positive CD8^+^ T cells significantly as well as the effector memory phenotype of cytokine-positive CD4^+^ T cells.

Comparing DNA vaccines expressing the mosaic Gag (Group 4) to those expressing the natural Gag (Group 3), cytokine-positive CD8+ T cells were 7-fold significantly higher in Group 4 (Gag^M^) than in Group 3 (Gag^N^) (Fig 3C; Group 4–1.12%; Group 3–0.16%; p<0.001). The effector memory phenotype in this T cell compartment was 100% and 84.5% in Group 4 and 3 mice respectively. Cytokine-production in the CD4+ T cell compartment was very similar for the two groups (Fig 3D; Group 4–0.38%; Group 3–0.31%). However, Group 4 mice had a lower proportion of cells that had an effector memory phenotype (76.7%).

Mice vaccinated with two doses of the DNA-Gag^M^ vaccine (Group 4) had predominantly CD8^+^ T cells responses, while those vaccinated with two doses of the MVA-Gag^M^ vaccine (Group 6) had predominantly CD4^+^ T cells responses (Fig 3C and 3D). Frequencies of cytokine-positive CD8^+^ T cells were 1.6-fold greater in Group 4 mice (1.12%) compared to Group 6 mice (0.71%). The proportion of cytokine-positive CD8^+^ T cells with an effector memory phenotype was also higher in Group 4 mice (100%) compared to Group 6 mice (91.6%). On the other hand, cytokine-positive CD4^+^ T cells were 2-fold greater in Group 6 mice (0.76%) compared to Group 4 mice (0.38%). The proportion of cytokine-positive CD4^+^ T cells with an effector memory phenotype was also higher in Group 6 mice (99%) compared to Group 4 mice (76.9%). As expected, two doses of MVA-Gag^M^ elicited higher frequencies of cytokine-producing T cells (Group 6) than a single dose (Group 5).

## Discussion

**Fig 4:**
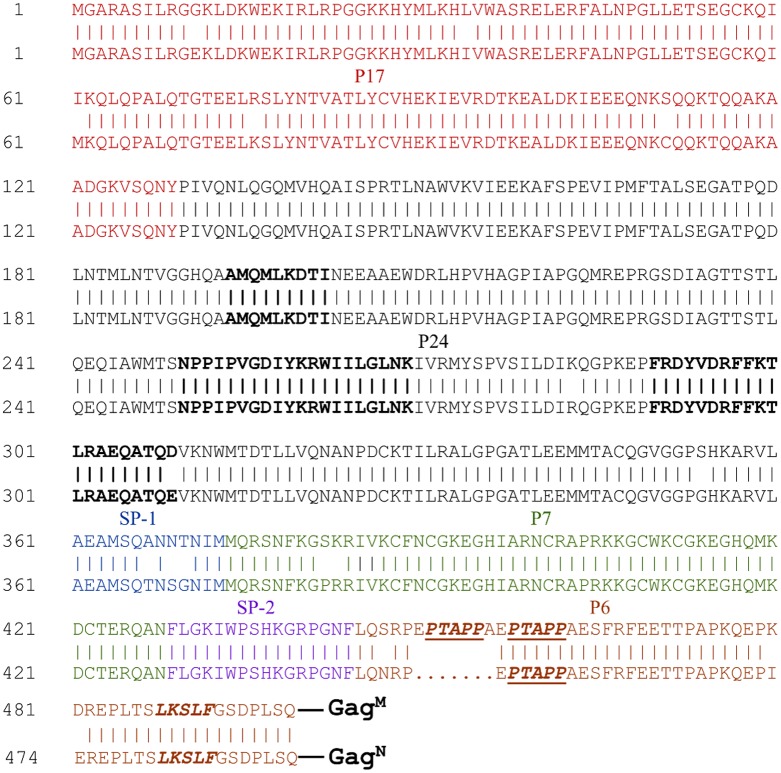
Amino acid sequence alignment of HIV-1 subtype C mosaic Gag and strain Du422 Gag. The P17 (matrix), P24 (capsid), SP-1(spacer region 1), P7 (nucleocapsid), SP-2 (spacer region 2) and P6 domains of Gag are shown. The PTAPP binding site for ESCRT-1 protein, TSG101, is indicated as is the LxxLF motif which interacts with ALIX protein of ESCRT-III. Mouse Gag CD8+ (AMQMLKDTI) and CD4+ (NPPIPVGRIYKRWIILGLNK GagCD4(13) and FRDYVDRFFKTLRAEQATQE GagCD4(17)) epitopes are indicated in black, bold font.

This study demonstrated that the HIV-1 subtype C mosaic Gag antigen (Gag^M^) generated T cell responses of greater magnitude and quality than a natural Gag (Gag^N^), in BALB/c mice. This was shown by directly comparing the immune responses after homologous vaccination of mice with DNA vaccines expressing these antigens. DNA-Gag^M^ homologous vaccination induced cumulative Gag-specific IFN-γ ELISPOT responses that were 6.5 fold higher than those induced by the DNA-Gag^N^ vaccination. Following DNA-Gag^M^ vaccination, the frequencies of cytokine-positive CD8+ and CD4+ T cells were 7 and 1.2 fold greater than DNA-Gag^N^. It is not clear why there is an improved immune response to the mosaic Gag as the amino acid sequences of Gag^M^ and Gag^N^ are very similar, the only major difference being a duplication of seven amino acids, EPTAPPA, in the p6 region (Fig 4). This PTAPP motif is known to bind to the TSG101 subunit of the endosomal sorting complex required for transport (ESCRT-I) which mediates budding of the virus from the cell [22]. A second motif found in p6, LxxLF, interacts with the ALIX protein which recruits ESCRT-III to the site enabling membrane fission and budding to occur [18]. However, the PTAPP motif seems to be the preferential means of release for most cell types. It is possible that having two PTAPP motifs enables the Gag^M^ VLPs to bud more rapidly than the Gag^N^ VLPs and this could then lead to an improved immune response.

The improved T cell responses generated by DNA-Gag^M^ may also be due to amino acid differences between the spacer 1 regions (SP1) of the Gag^M^ and Gag^N^ (Fig 4). This region and the C terminus of the capsid protein of Gag have been shown to be involved in virus particle assembly and contribute to tetramerization of Gag. Intermolecular interactions in these regions are thought to stabilize the Gag lattice [23]. Thus differences in the SP1 region could lead to differences in the stability or structure of the VLPs leading to the exposure of different regions of the protein to the immune system. Therefore, although similar amounts of p24 Gag were expressed by cells transfected with DNA-Gag^M^ and DNA-Gag^N^ over time (Fig 1A), the VLPs produced by Gag^M^ may be more stable.

The mouse CD4+ and CD8+ T cell epitope sequences were identical in Gag^M^ and Gag^N^ with the exception of the Gag CD4(17) epitope which differs in the last amino acid (Gag^M^–FRDYVDRFFKTLRAEQATQD and Gag^N^–FRDYVDRFFKTLRAEQATQE). Flanking sequences have been shown to impact epitope processing but the sequences flanking epitopes did not differ between the two VLPs [24].

DNA prime, poxvirus boost regimens have been shown to give potent HIV-1-specific immune responses that are better than those generated with homologous vaccination regimens [25–27]. In the EV02 phase I clinical trial one group of volunteers received two injections consisting of two plasmids expressing a GagPolNef polyprotein or gp120 followed by two injections of NYVAC expressing identical immunogens [11]. The second group only received two injections of NYVAC. Volunteers in the DNA prime NYVAC boost group had higher and more durable IFN-γ ELISPOT responses as compared to the group that only received NYVAC. There were also more responders in the DNA prime group with 18/20 responding (90%), whilst only 6/15 (40%) responded in the NYVAC alone group. Similarly, our data indicated a heterologous DNA-Gag^M^ prime, MVA-Gag^M^ boost was more immunogenic than either a DNA-Gag^M^ or MVA-Gag^M^ homologous prime boost. The heterologous DNA-Gag^M^ prime, MVA-Gag^M^ boost was also more immunogenic than a BCG-Gag^M^ prime, MVA-Gag^M^ boost (15). Two DNA-Gag^M^ primes followed by an MVA-Gag^M^ boost elicited a 2.3 fold higher IFN-γ ELISPOT response than a single BCG-Gag^M^ prime MVA-Gag^M^ boost and a 2.2 fold higher percentage of cytokine positive CD8+ T cells.

T cell responses in the EV02 trial were mainly of the CD4+ phenotype, primarily against Env and were of higher magnitude than those against Gag, Pol & Nef [28]. This is not ideal as Gag-specific CD8+ T cell responses have been found to be associated with lower viral loads in long term non-progressors, elite controllers and individuals that have been exposed to HIV but are seronegative. Env-specific T cell responses have been associated with poor viral control [29]. The vaccines used in the EV02 trial have subsequently been modified by increasing expression of Gag, reintroducing a myristoylation signal to allow formation of GagPolNef VLPs, replacing gp120 with gp140 and expressing the proteins in different vectors [30]. When tested in macaques the modified vaccines generated immune responses of greater magnitude, breadth and quality than those used in the EV02 trial. The vaccines used in our study contain all these modifications: the Gag is myristoylated, has been shown to form VLPs and is expressed at high levels from both DNA and MVA vectors.

The high immune responses observed in the DNA-Gag^M^/MVA-Gag^M^ heterologous vaccination regimen could be attributable to the increased and early levels of Gag^M^ expression by MVA-Gag^M^. Furthermore, the ability of MVA-Gag^M^ to form VLPs may also be associated with the potent immune responses. VLPs can stimulate the immune system better than antigens that are not particulate. It has been shown that immune responses induced by a DNA-based HIV-1 vaccine can be elevated if the expressed antigen forms VLPs in vitro or is co-administered with HIV-1 VLPs [31, 32].

Phase I and II clinical studies using DNA and MVA-based HIV vaccines expressing natural immunogens have been conducted with very promising results. In addition, we have demonstrated in a nonhuman primate study (Chege GK et al, in press) and a Phase 1 clinical trial [14] the potential of further improvement on the immunogenicity of these vaccines by adding a HIV-1 gp140 Env protein boost to a DNA-MVA regimen, resulting in generation of functional antibodies capable of neutralizing multiple Tier 1 viruses and mediating ADCC activity. Here, we show that DNA and MVA vaccines expressing HIV-1 subtype C mosaic Gag are more immunogenic in mice than those expressing natural immunogens and warrant further investigation in non-human primates.
